# Jupyter notebook-based tools for building structured datasets from the Sequence Read Archive

**DOI:** 10.12688/f1000research.23180.2

**Published:** 2020-08-04

**Authors:** Matthew N. Bernstein, Ariella Gladstein, Khun Zaw Latt, Emily Clough, Ben Busby, Allissa Dillman

**Affiliations:** 1 Morgridge Institute for Research, Madison, Wisconsin, 53715, USA; 2 Department of Genetics, University of North Carolina at Chapel Hill, Chapel Hill, North Carolina, 27599, USA; 3 Kidney Disease Branch, National Institute of Diabetes and Digestive and Kidney Diseases, Bethesda, Maryland, 20892, USA; 4 National Center for Biotechnology Information NLM, Bethesda, Maryland, 20894, USA

**Keywords:** Hackathon, RNA-seq, Sequence Read Archive, MetaSRA, Metadata, Ontology, Jupyter

## Abstract

The Sequence Read Archive (SRA) is a large public repository that stores raw next-generation sequencing data from thousands of diverse scientific investigations.  Despite its promise, reuse and re-analysis of SRA data has been challenged by the heterogeneity and poor quality of the metadata that describe its biological samples. Recently, the MetaSRA project standardized these metadata by annotating each sample with terms from biomedical ontologies. In this work, we present a pair of Jupyter notebook-based tools that utilize the MetaSRA for building structured datasets from the SRA in order to facilitate secondary analyses of the SRA’s human RNA-seq data. The first tool, called the *Case-Control Finder*, finds suitable case and control samples for a given disease or condition where the cases and controls are matched by tissue or cell type.  The second tool, called the *Series Finder*, finds ordered sets of samples for the purpose of addressing biological questions pertaining to changes over a numerical property such as time. These tools were the result of a three-day-long NCBI Codeathon in March 2019 held at the University of North Carolina at Chapel Hill.

## Introduction

The Sequence Read Archive (SRA; Leinonen *et al*., 2011) is a large public repository that stores next-generation sequencing data from thousands of diverse scientific investigations. Despite its promise, reuse and re-analysis of SRA data has been challenged by the heterogeneity and poor quality of the metadata that describe its biological samples ( Gonçalves & Musen, 2019). Recently, the MetaSRA project ( Bernstein *et al*., 2017) standardized these metadata by annotating each sample with terms from biomedical ontologies including Cell Ontology ( Bard *et al*., 2005), Uberon ( Mungall *et al*., 2012), Disease Ontology ( Schriml *et al*., 2019), Cellosaurus ( Bairoch, 2018), and the Experimental Factors Ontology ( Malone *et al*., 2010). The MetaSRA also features an interface ( http://metasra.biostat.wisc.edu) for querying human RNA-seq samples using these ontology term annotations. However, the MetaSRA web interface is not capable of producing structured datasets such as those that match case samples associated with a target condition or disease with healthy control samples. Similarly, the MetaSRA is also not capable of searching for samples associated with a particular condition and/or tissue-type that are ordered according to a numeric property (e.g., age).

Construction of such datasets is non-trivial and requires further processing of the results provided by the MetaSRA website. Specifically, finding case and control samples for a given disease requires matching case samples to control samples according to their tissue or cell type. For example, if one were to naively search the MetaSRA for “liver cancer” samples, the results would include samples from Kim *et al.* (2020), which consist of isolated T cells from liver tumors. Therefore, only matched T cell samples would make for appropriate controls. Furthermore, given these search results, users may wish to further filter samples according to whether they are poorly annotated (i.e. , are missing cell type or tissue information), whether they are derived from a cell line, or whether they were experimentally treated. Moreover, given these results, the user may wish to explore other ontology terms associated with the search results within either the case or control samples to check for any variables that may confound downstream analyses. Finding longitudinal or time-series data presents similar challenges. To the best of our knowledge, no existing tool addresses these tasks.

To address these two tasks, we produced two Jupyter notebook-based tools. The first tool, called the *Case-Control Finder*, searches the SRA via the MetaSRA terms to produce matched-case and control samples for a given disease or condition where the cases and controls are matched by tissue and cell type. The second tool, called the *Series Finder*, finds ordered sets of samples for the purpose of answering biological questions pertaining to changes over a numerical property (e.g., time). More specifically, the Series Finder produces ordered sets of samples, where the order is determined based on a temporal property in the metadata as standardized by the MetaSRA’s real-valued properties. Examples of temporal properties include the age of a person from which a given sample originated or the time in which a given sample of cells have spent differentiating *in vitro*. These tools promise to facilitate the construction of suitable public datasets for secondary analyses.

## Methods

The tools presented in this work were written in Python (v3.6) and make use of Python packages pandas ( McKinney, 2011), Matplotlib ( Hunter, 2007), and seaborn ( https://seaborn.pydata.org). These notebooks can be run in the cloud via Google Colab. A link to these notebooks can be found in the README within the Github repository ( https://github.com/mbernste/hypothesis-driven-SRA-queries).

### Case-Control Finder

**Figure 1.**
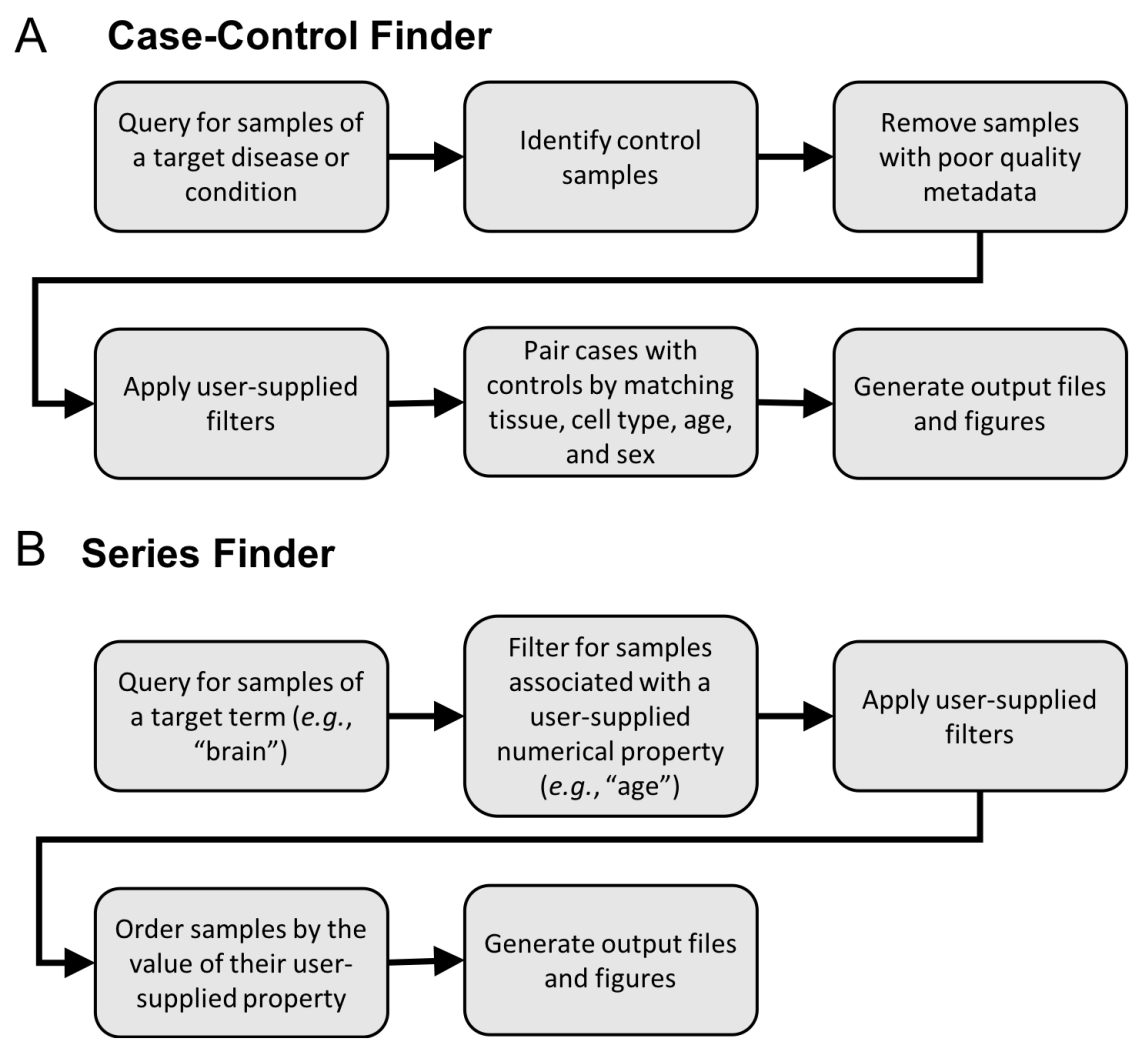
Data flows for hypothesis-driven query tools. An overview of the backend processing functions called from the Jupyter notebooks.

**Figure 2.**
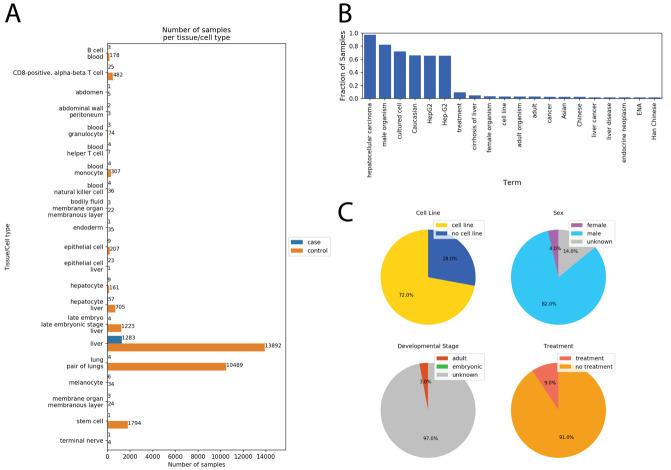
Example results from the Case-Control Finder. Results from running the Case-Control Finder for the query “liver cancer.” ( **A**) The Case-Control Finder displays the number of case/control samples matched by each tissue and cell type. ( **B**) The user can select either the case samples or control samples for a given tissue or cell type and display the most common ontology terms associated with those selected samples. Displayed here are the most common terms associated with the case samples labeled as “liver.” ( **C**) The notebook also displays four pie charts for viewing the fraction of samples belonging to a cell line (top left), each sex (top right), each developmental stage (bottom left), and whether they were given an experimental treatment (bottom right).

The Case-Control Finder implements the following steps to produce a dataset of matched-case control samples for a given disease ( Figure 1A):

1. **Generate candidate case and control samples.** Generate the set of candidate case samples by querying for all samples associated with a user-specified condition or disease using the MetaSRA-mapped ontology terms. Also, find all candidate control samples that are *not* associated with the target condition/disease.
2. **Filter poorly annotated samples.** Filter samples based on a metadata completeness threshold, which requires that all samples be associated with either a tissue term or a cell type term. The tissue/cell type information is required for downstream matching of case samples to control samples.
3. **Apply user-specified filters.** Further filter samples according to user-specified filtering parameters. The user can filter out cell line samples, treated samples, and *in vitro* differentiated samples. The user can also remove all diseased samples from the candidate control samples for the purpose of generating a healthy control-set.
4. **Match by tissue, cell type, age, and sex.** The candidate case samples are then matched with the candidate control samples by their tissue and cell type terms. Optionally, the user can also match samples by age and sex. Specifically, given that each sample can be associated with multiple ontology terms in the MetaSRA, a set of case samples is matched with a set of control samples when both sets of samples are labelled with the same set of tissue and cell type terms. For example, a set of case samples annotated with the set of terms “liver” and “epithelial cell” will be matched only to control samples also labeled strictly with these terms ( Figure 2A). This ensures that case samples are matched with maximally similar control samples and mitigates matching samples from different tissue-types. For example, a set of case samples labelled with both the terms “liver” and “epithelial cell” will not be matched with a set of samples labelled only as “epithelial cell,” as there is no guarantee that the latter set of samples originate in the liver.

Once the dataset is constructed, the notebook enables the user to explore the samples for other MetaSRA mapped ontology terms within the data ( Figure 2B and C). By presenting other common ontology terms in the data, the user may be able to identify variables that either confound analysis.

### Series Finder

The Series Finder finds RNA-seq data samples that are associated with a numerical property (e.g., age or time point) for a given tissue or cell type. To do so, the Series Finder utilizes the real-value property annotations provided by the MetaSRA where each real-value property in the MetaSRA is structured as a tuple consisting of a property name (e.g., age), numerical value, and unit (e.g., year).

To perform a query, the user provides an ontology term, such as a tissue or cell type, as well as a property name and unit. The Series Finder then finds all samples that are associated with the target ontology term and real-value property. The user can also specify a set of filters (e.g. for filtering diseased samples or cell line samples) and the Series Finder will remove all samples that meet the filter specification. The Series Finder will then return all remaining samples ordered by their associated numerical values ( Figure 1B).

## Results and use cases

We used the Case-Control Finder to query for samples of liver cancer RNA-seq samples matched with healthy control samples. This query resulted in 21 sets of samples representing different tissues or cell types including epithelial cells, hepatocytes, stem cells, and liver tissue ( Figure 2A). The Case-Control Finder identified common terms associated with the case “liver cancer” samples ( Figure 2B), and categorized these samples by cell line status, sex, developmental stage, and treatment status ( Figure 2C).

**Figure 3.**
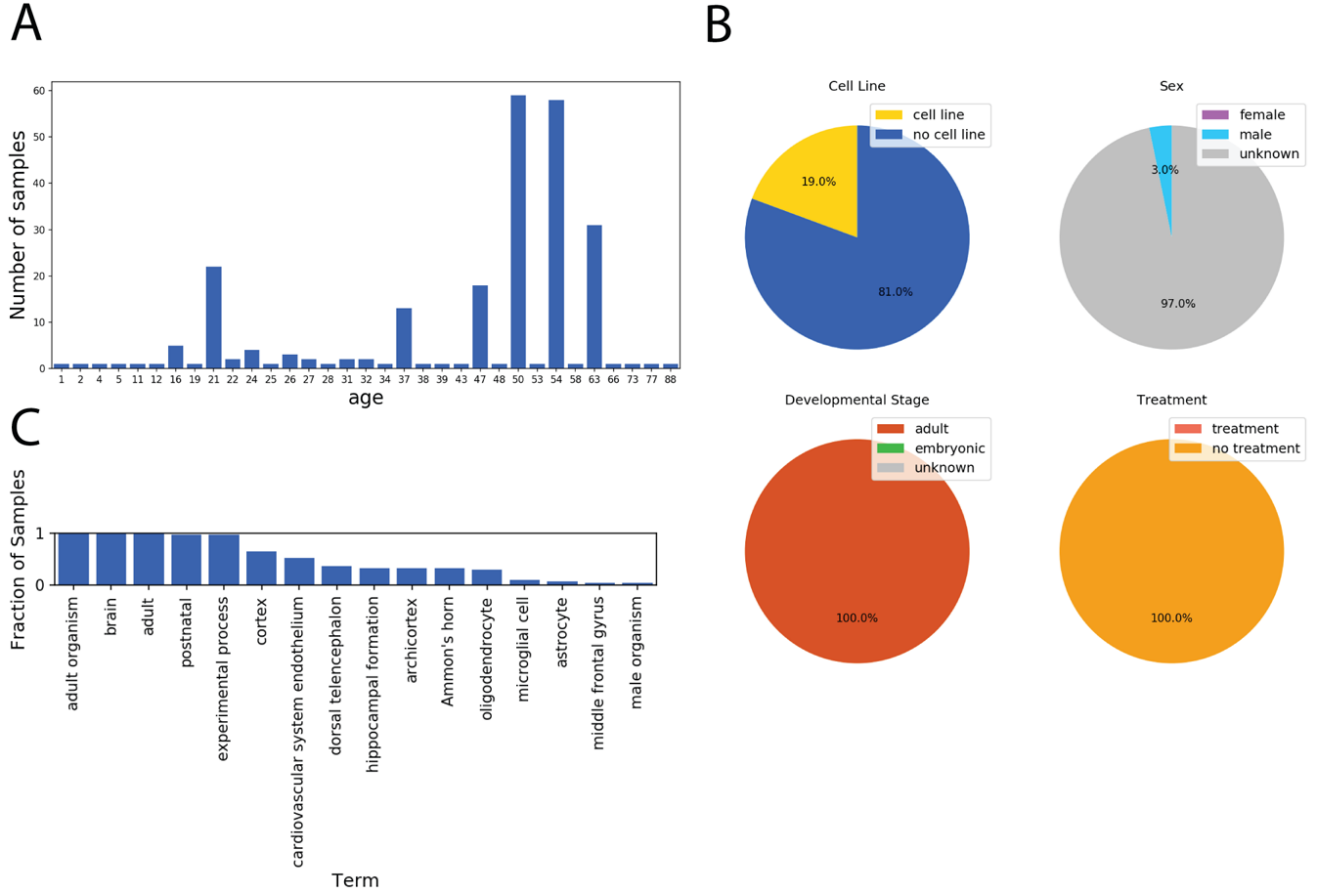
Example results from the Series Finder. Results from running the Series Finder for the query “brain” sorted by “age,” where unit is specified as “year.” ( **A**) The Series Finder displays the number of samples sorted by age. ( **B**) The user can select samples associated with a given time point for further exploration. Here the samples annotated as “year = 63” are selected. The notebook then displays four pie charts for viewing the fraction of samples belonging to a cell line (top left), each sex (top right), each developmental stage (bottom left), and whether they were given an experimental treatment (bottom right). ( **C**) Given the selected samples from ( **B**), the notebook displays the most frequent terms associated with those selected samples.

We used the Series Finder to find all brain samples in the SRA ordered by the age of the sample donor. This query resulted in samples spanning many ages ( Figure 3A). This dataset could prove useful for exploring gene expression-based signatures of aging. The Series Finder also identified common terms at each age ( Figure 3B) and for each age’s sample-set, categorized those samples by cell line status, sex, developmental stage, and treatment status ( Figure 3C).

## Conclusion and future work

We implemented two Jupyter notebooks for performing hypothesis-driven queries of public RNA-seq samples in the SRA. These tools are built upon the standardized metadata provided by the MetaSRA project and enable querying of the metadata beyond what is natively possible via the MetaSRA website interface. Given the SRA accessions of the RNA-seq samples that these tools produce, a user can then retrieve the gene expression data for these samples in order to perform secondary analyses. Specifically, the user can either download and process the raw reads from the SRA, or they can obtain preprocessed gene expression profiles from recent mass preprocessing efforts such as recount2 ( Collado-Torres, 2017), ARCHS4 ( Lachmann *et al*., 2018), and refine.bio ( Greene *et al*.). Finally, these notebooks come pre-packaged with metadata files from the latest version of the SRA, as provided by the SRAdb ( Yuelin *et al*., 2013), and MetaSRA. When the MetaSRA releases a new version of annotated metadata, these notebooks will be updated to track the new release. 

We also note a few limitations to this work. First given that the MetaSRA annotates the SRA samples using an automated computational pipeline, its annotations contain some errors. Errors in the MetaSRA may propagate to the results produced by these tools, and thus, the datasets produced by these tools are best utilized as sets of *candidate* datasets for downstream analysis. We point the reader to Bernstein *et al*. (2017) for an analysis of the MetaSRA’s accuracy. We also note that the SRA stores sequencing data for both bulk RNA-seq and single-cell RNA-seq samples; however, this information is not encoded in any standardized way within the SRA nor is it captured by the MetaSRA. Thus, results returned by these tools may include a mixture of both single-cell and bulk data. For these reasons, we encourage users to validate the results returned by these tools by consulting their entries in the SRA before proceeding with downstream analyses. Lastly, to facilitate access to these tools, it would benefit to implement them within an easy-to-use web interface rather than Jupyter notebooks. Future work will entail either integrating these tools into the MetaSRA website, or implementing a stand-alone web application for these tools using a platform such as R Shiny.

## Data availability

The figures and datasets produced in the analyses can be found on GitHub: https://github.com/mbernste/hypothesis-driven-SRA-queries/tree/master/results

## Software availability

All code is maintained on GitHub: https://github.com/mbernste/hypothesis-driven-SRA-queries

Archived code as at time of publication: https://doi.org/10.5281/zenodo.3957949 ( Bernstein, 2020)

License: CC0
