# Supplementary material for: Jupyter notebook-based tools for building structured datasets from the Sequence Read Archive
Source: F1000Res. 2020 Aug 4;9:376. Originally published 2020 May 19. [Version 2] doi: 10.12688/f1000research.23180.2 (PMC7445559; doi:10.12688/f1000research.23180.2)
Supplement: Peer review history

# Peer review: Reviewer response for version 2

**DOI:** 10.5256/f1000research.27241.r68621

**Published:** 2020-08-24

**Authors:** Zichen Wang

**Affiliations:** 1 Sema4, Stamford, CT, USA

The authors have addressed all of my comments and significantly improved the tools.

Are the conclusions about the tool and its performance adequately supported by the findings presented in the article?

Partly

Is the rationale for developing the new software tool clearly explained?

Yes

Is the description of the software tool technically sound?

Yes

Are sufficient details of the code, methods and analysis (if applicable) provided to allow replication of the software development and its use by others?

Yes

Is sufficient information provided to allow interpretation of the expected output datasets and any results generated using the tool?

Partly

Reviewer Expertise:

Bioinformatics; Computational Biology; Machine Learning

I confirm that I have read this submission and believe that I have an appropriate level of expertise to confirm that it is of an acceptable scientific standard.

# Peer review: Reviewer response for version 2

**DOI:** 10.5256/f1000research.27241.r68620

**Published:** 2020-08-12

**Authors:** Shannon Ellis

**Affiliations:** 1 Department of Cognitive Science, UC San Diego, La Jolla, CA, USA; 2 Department of Biostatistics, Johns Hopkins University School of Public Health, Baltimore, MD, USA

Thank you to the authors for their thoughtful response and changes to the manuscript & notebooks. I'm happy with the current version of the manuscript and associated materials.

Are the conclusions about the tool and its performance adequately supported by the findings presented in the article?

Partly

Is the rationale for developing the new software tool clearly explained?

Yes

Is the description of the software tool technically sound?

Yes

Are sufficient details of the code, methods and analysis (if applicable) provided to allow replication of the software development and its use by others?

Yes

Is sufficient information provided to allow interpretation of the expected output datasets and any results generated using the tool?

Partly

Reviewer Expertise:

genetics, bioinformatics, data science education

I confirm that I have read this submission and believe that I have an appropriate level of expertise to confirm that it is of an acceptable scientific standard.

# Peer review: Reviewer response for version 1

**DOI:** 10.5256/f1000research.25586.r63614

**Published:** 2020-06-05

**Authors:** Shannon Ellis

**Affiliations:** 1 Department of Cognitive Science, UC San Diego, La Jolla, CA, USA; 2 Department of Biostatistics, Johns Hopkins University School of Public Health, Baltimore, MD, USA

This paper describes the development of two Jupyter notebook-based tools (Case-Control Finder and Series Finder) for improving the ease with which researchers can identify cases within the SRA for further study.

 While the paper does a nice job describing what the tool is and how it can be helpful and the code & examples provided/explained the paper function as expected (is reproducible), there are a few limitations in its implementation that will limit its utility with researchers:

  

1. The fact that this tool requires a static version of the SRA metadata to be loaded in limits its ability to be updated and requires the authors to manually download the metadata - access by API to SRA would improve this process.
2. While the provided examples work well, there are limitations to unfamiliar users and failures in cases that seem on reading the paper like they should work.
  - For example: in series finder if I change `term` to "heart" (instead of "brain"), almost all subsequent cells fail.
  - In case-control finder, if I change `condition` to "brain cancer", all but one samples returned are controls (which does not align with what is in the SRA?) and visualization formatting becomes difficult.
  - By clarifying what user options are (or examples) for each place where user is free to play with the input, this could be avoided. Similarly, functions lack documentation and examples here or checks on input within the functions, so diving into the code becomes critical for use, which will limit users. Adding documentation and checks for user input could assist in this overall.

Minor issues:

1. I was able to download locally using the "not recommended" approach; however, docker asked for a password using suggested approach in README (I didn't investigate further).
2. In the paper & notebooks, tool would be improved by focusing on readability of visualizations. For example, flipping the bar charts in figure 2A by 90 degrees (and accompanying in the notebook), the labels would be more readable. And, by considering the colors in figure C, such that "orange" is not used in all three pie charts (when they do not represent the same categories) would be helpful. Having the number of samples summarized by the pie charts would also be helpful.
3. The sentence in Introduction starting with "More specifically, the Series Finder produces..." is unclear. Specifically, on reading, I'm not sure what a temporal property would be in the metadata (other than the listed age). As a reader, this limits my understanding of 1 of the two notebooks provided and my ability to use the tool.
4. I may be missing it, but it seems like cases and controls would benefit most from being able to also be matched on age and sex to truly make them useful for further analysis. It does not seem this functionality exists, or I'm missing it.

Are the conclusions about the tool and its performance adequately supported by the findings presented in the article?

Partly

Is the rationale for developing the new software tool clearly explained?

Yes

Is the description of the software tool technically sound?

Yes

Are sufficient details of the code, methods and analysis (if applicable) provided to allow replication of the software development and its use by others?

Yes

Is sufficient information provided to allow interpretation of the expected output datasets and any results generated using the tool?

Partly

Reviewer Expertise:

genetics, bioinformatics, data science education

I confirm that I have read this submission and believe that I have an appropriate level of expertise to confirm that it is of an acceptable scientific standard, however I have significant reservations, as outlined above.

# Response

**Published:** 2020-07-23

**Authors:** Matthew Bernstein

**Affiliations:** Morgridge Institute for Research, USA

We greatly appreciate the reviewer's valuable suggestions and feedback. Please see our responses below:

1. We agree that using the MetaSRA’s API would be a great idea; however, the API restricts queries that return too many results. Specifically, for queries that return too many results, the API returns an error message that the search results are too large. This severely restricts our ability to use the API for these tools. We note that the MetaSRA is released in discrete chunks and does not track every ongoing change to the SRA; thus, whenever the MetaSRA version changes, we will update the static version of the MetaSRA packaged with these tools. We have added text to this manuscript detailing our commitment to performing these updates. Lastly, we added text to the README that makes it more explicit to the user which version of the MetaSRA these tools are utilizing.

2. 

- We tested the query “heart” and it now should return results. We also provide more thorough input validation for cases in which the query does not return results.

- We have updated the code so that the tools retrieves sample that are annotated as an ancestral term to the query term (e.g. a sample labelled as “brain glioma” should be retrieved when the user inputs the query “brain cancer”). Now the query “brain cancer” will retrieve many more samples than before. We do note a few issues with the particular query “brain cancer” (which maps to term DOID:1319 in the Disease Ontology).  Specifically, we found that the MetaSRA failed to label many samples as “brain cancer” due to the fact that many of the subterms (e.g. “brain glioma”) are missing important synonyms that would have led the MetaSRA to pick them up. For example, the term “brain glioma” (DOID:0060108) is not associated with the simple synonym “glioma” and thus, unless a sample for a given glioma sample was described using the string “brain glioma”, which appears to be rare, the MetaSRA failed to annotate this sample as a “brain glioma”.  Instead, the MetaSRA labels glioma samples using an alternative “glioma” term from the Experimental Factors Ontology (EFO:0005543), which does not have “brain cancer” as an ancestor term, but instead has “brain neoplasm” as an ancestor (EFO:0003833). This case points to the fact that there is still work to be done in both standardizing the metadata in the SRA and in constructing comprehensive ontologies. Unfortunately, these issues remain out of the scope for this work; however, we now include new text in the Conclusion section that discusses how the original MetaSRA annotations contain some errors and that these errors may propagate to the output of these tools. 

- Thank you for this suggestion. We have added more detailed instructions for each input parameter. We also perform more thorough input-validation on the user’s input. Lastly, we have added more documentation to each function in utils to help a user who wishes to dive further into the code.

Responses to minor issues:

1. We apologize for this password issue. Given how few dependencies these notebooks utilize, we decided that Docker is probably overkill for this project and therefore we removed this option altogether. We instead uploaded these notebooks to Google Colab to run in the cloud.  If a user would like to run the notebooks locally, we now detail all of the dependencies in the file “requirements.txt” within the repository and offer guidance on installing these dependencies in the README. 

2. Thank you for these suggestions. We flipped the barcharts 90 degrees and also use a different color palette for each pie chart. We note that the same samples are used to construct each of the four pie charts.

3. We added text to this sentence highlighting another example of a temporal property: time in which cells have spent differentiating in vitro. To this end, we have also added another parameter to the query that enables users to select only in vitro differentiating cells in order to answer possible biological questions pertaining to differentiation.

4. This is definitely an important feature, thank you for suggesting it. We now enable the user to match by age and sex in the notebook (see Section “3. Set filtering parameters”) in the notebook. Specifically, in the notebook, if the user sets the variable “MATCH_BY_SEX” to True, we only consider samples that are annotated by sex in the MetaSRA and then match accordingly.  Similarly, if the user sets “MATCH_BY_AGE” to True, we only consider samples that are annotated with age and then match accordingly.

# Peer review: Reviewer response for version 1

**DOI:** 10.5256/f1000research.25586.r63613

**Published:** 2020-06-01

**Authors:** Zichen Wang

**Affiliations:** 1 Sema4, Stamford, CT, USA

Bernstein *et al.* provides two Jupyter notebook-based tools to facilitate re-analysis of human RNA-seq data deposited to SRA. The tools were built on top of annotated metadata of RNA-seq samples from the MetaRNA, and provided some visualizations of the summary statistics of the query results.

 I have the following suggestions and comments:

1. The authors should indicate how to access the Jupyter notebooks in the abstract.
2. It would require less overhead for users if the authors make their Jupyter notebook tools available to execute on Binder or Google Colab.
3. Since MetaSRA mapped RNA-seq samples to biomedical ontologies, it would be useful to have the Jupyter tools also enable query using ontology terms in addition to free texts. For instance, a researcher may want to focus on samples from non-small cell lung carcinoma (DOID:3908) rather than any types of lung cancers.
4. Currently, both notebooks load the metadata of the SRA samples from a preprocessed file in the Git repository. It would be useful to make it interoperable with MetaSRA through API to be able to query against the most updated version of SRA, which may include many more samples. As the volume of public RNA-seq data are drastically increasing.
5. Please provide available options for the structured query, including "target_property" and "UNIT", in the "Series Finder" notebook.
6. Please provide assessment of the precision and recall of the tools in terms of retrieving the correct samples given queries.  
7. Can the authors please comment on the applicability of the tools on bulk vs. single-cell samples?
8. Please add discussion about how to perform secondary analysis on the SRA samples after obtaining the structured data from the Jupyter notebooks.

Are the conclusions about the tool and its performance adequately supported by the findings presented in the article?

Partly

Is the rationale for developing the new software tool clearly explained?

Yes

Is the description of the software tool technically sound?

Yes

Are sufficient details of the code, methods and analysis (if applicable) provided to allow replication of the software development and its use by others?

Yes

Is sufficient information provided to allow interpretation of the expected output datasets and any results generated using the tool?

Partly

Reviewer Expertise:

Bioinformatics; Computational Biology

I confirm that I have read this submission and believe that I have an appropriate level of expertise to confirm that it is of an acceptable scientific standard, however I have significant reservations, as outlined above.

# Response

**Published:** 2020-07-23

**Authors:** Matthew Bernstein

**Affiliations:** Morgridge Institute for Research, USA

We greatly appreciate the reviewer's valuable feedback. Please find our responses to each point below:

1. Within the abstract we now point the reader to the tools’ Github repository, which describes how the tools can be executed either locally or in the cloud via Google Colab.

2. We have set up Google Colab notebooks to run these tools in the cloud. Links to the notebooks are found within the README in the Github repository.

3. We thank you for this suggestion. We have updated the tools to now accept both ontology term names (i.e. free text) as well as ontology term ID’s.

4. We agree that using the MetaSRA’s API would be a great idea; however, the API restricts queries that return too many results. Specifically, for queries that return too many results, the API returns an error message that the search results are too large. This severely restricts our ability to use the API for these tools.  We note that the MetaSRA is released in discrete chunks and does not track every ongoing change to the SRA; thus, whenever the MetaSRA version changes, we will update the static version of the MetaSRA packaged with these tools. We have added text to this manuscript detailing our commitment to performing these updates. Lastly, we added text to the README that makes it more explicit to the user which version of the MetaSRA these tools are utilizing.

5. Within the instructions (within Section 1 of the Series Finder), we  now provide the user example properties (such as “passage number” and “time”) as well as example units (such as “hour” and “day”). We also point the user to the Units Ontology for a full set of available units that are utilized by the underlying MetaSRA annotations.

6. We note that the accuracy of the results is dependent on the accuracy of the MetaSRA annotations, which have been thoroughly evaluated in  the original MetaSRA publication by Bernstein et al. (2017). Therefore, we added text to the “Conclusion and future work” section that points readers to this analysis.  We have also added text to this section that clarifies that these tools are for selecting an initial *candidate* set of samples from the SRA; however, given that the annotations are not error-free, we encourage the user to further validate the datasets returned by these tools before performing downstream analysis.

7. The SRA stores sequencing data for both bulk and single-cell data; however, this information is not encoded in the metadata in a standardized way nor is it captured by the MetaSRA.  Therefore, one limitation of the tools presented in this work is that they may return datasets that comprise both bulk and single-cell samples.  We describe this limitation in the Conclusion section and again encourage users to validate the results returned by these tools before performing downstream analyses.

8. In the Conclusion section, we now point the reader to databases of pre-processed SRA data including recount2, ARCHS4, and refine.bio.  From these resources, users can download pre-processed expression data for the samples returned by the tools presented in this work.
